# Karyology and Genome Size Analyses of Iranian Endemic *Pimpinella* (Apiaceae) Species

**DOI:** 10.3389/fpls.2022.898881

**Published:** 2022-06-15

**Authors:** Shaghayegh Mehravi, Gholam Ali Ranjbar, Hamid Najafi-Zarrini, Ghader Mirzaghaderi, Mehrdad Hanifei, Anita Alice Severn-Ellis, David Edwards, Jacqueline Batley

**Affiliations:** ^1^School of Biological Sciences, University of Western Australia, Perth, WA, Australia; ^2^Department of Plant Breeding and Biotechnology, Faculty of Crop Sciences, Sari Agricultural Sciences and Natural Resources University, Sari, Iran; ^3^Department of Agronomy and Plant Breeding, Faculty of Agriculture, University of Kurdistan, Kurdistan, Iran; ^4^Department of Plant Genetics and Breeding, Faculty of Agriculture, Tarbiat Modares University, Tehran, Iran

**Keywords:** *Pimpinella*, chromosome, karyology, DNA *C*-value, genome size, B-chromosome

## Abstract

*Pimpinella* species are annual, biennial, and perennial semibushy aromatic plants cultivated for folk medicine, pharmaceuticals, food, and spices. The karyology and genome size of 17 populations of 16 different *Pimpinella* species collected from different locations in Iran were analyzed for inter-specific karyotypic and genome size variations. For karyological studies, root tips were squashed and painted with a DAPI solution (1 mg/ml). For flow cytometric measurements, fresh leaves of the standard reference (*Solanum lycopersicum* cv. Stupick, 2C DNA = 1.96 pg) and the *Pimpinella* samples were stained with propidium iodide. We identified two ploidy levels: diploid (2*x*) and tetraploid (4*x*), as well as five metaphase chromosomal counts of 18, 20, 22, 24, and 40. 2*n* = 24 is reported for the first time in the *Pimpinella* genus, and the presence of a B-chromosome is reported for one species. The nuclear DNA content ranged from 2C = 2.48 to 2C = 5.50 pg, along with a wide range of genome sizes between 1212.72 and 2689.50 Mbp. The average monoploid genome size and the average value of 2C DNA/chromosome were not proportional to ploidy. There were considerable positive correlations between 2C DNA and total chromatin length and total chromosomal volume. The present study results enable us to classify the genus *Pimpinella* with a high degree of morphological variation in Iran. In addition, cytological studies demonstrate karyotypic differences between *P. anthriscoides* and other species of *Pimpinella*, which may be utilized as a novel identification key to affiliate into a distinct, new genus – *Pseudopimpinella*.

## Introduction

The genus *Pimpinella* is one of the largest genera in the family Apiaceae, subfamily Apioideae, and tribe Pimpinelleae, with approximately 170–180 species distributed throughout Europe, Asia, Africa, and South and North America (Pimenov and Leonov, 1993; Pu and Watson, 2005). The members of this genus are annual, biennial or perennial, semibushy, and aromatic plants with cordate-ovoid or oblong-ovoid fruits with five filiform ribs on each cordate-ovoid or oblong-ovoid leaves (Pu and Watson, 2005). Nearly 23 species of this genus are grown in Iran, six of which are endemic (*P. pastinacifolia*, *P. tragioides*, *P. khorasanica*, *P. deverroides*, *P. khayyamii*, and *P. anisactis*) (Mozaffarian, 2003). Their habitats are dry slopes, rocky crevices, fields, meadows, mountain pastures, grasslands, steppes, and dry open woodlands 1,000–2,200 m above sea level.

Studies suggest that the genus *Pimpinella* is highly diverse, and the taxonomic delimitation of the genus has not yet been resolved (Zhou et al., 2008, 2009; Downie et al., 2010). The last revision of the genus was made by Wolff (1927) based on the petal color, fruit and petal vestiture, and life history. The genus was subdivided into three sections: *Reutera*, *Tragium*, and *Tragoselinum*. It has since been realized that morphological markers do not explain the systematic relationship among *Pimpinella* species (Fereidounfar et al., 2016). Hence, investigation of various aspects, including karyological observations and genome size estimates, may be useful in establishing systematic and evolutionary relationships, resolving taxonomic ambiguities, and gaining a better understanding of the way they diverged from each other (Dobigny et al., 2004; Knight et al., 2005; Bancheva and Greilhuber, 2006; Guerra, 2008; Bainard et al., 2013).

Basic chromosome number (*x*) variations of *Pimpinella* species have been shown by previous karyotypic studies as follows: *x* = 8 for *P. affinis* (Yurtseva, 1988), *x* = 9 for *P. tragium*, *P. saxifrage* and *P. puberula* (Gawlowska, 1967; Yurtseva, 1988; Pimenov et al., 2003), *x* = 10 for *P. corymbosa* and *P. lutea* (Al-Eisawi, 1989; Verlaque and Filosa, 1992; Pimenov et al., 1996), *x* = 11 for *P. buchananii*, *P. trifurcate*, and *P. tragium* (Constance and Chuang, 1982; Abebe, 1992; Yurtseva and Tikhomirov, 1998; Pimenov et al., 2003) and *x* = 18 and 20 for *P. saxifrage* (Gawlowska, 1967). However, a literature review suggests that *x* = 9 is the plesiomorphic state within the *Pimpinella*, which – with subsequent aneuploidy – resulted in an increase to *x* = 10, 11, and 12 in the more derived species, which seems to be a plausible hypothesis. For *Pimpinella*, currently, the most accepted basic chromosome number is *x* = 9. Polyploidy and aneuploidy levels have also been reported for *Pimpinella* species (Constance and Chuang, 1982; Daushkevich et al., 1995; Shner et al., 2004). Overall, the extraordinary variations in *Pimpinella* chromosome number can be reflected in inter/intraspecific nuclear DNA contents. Nuclear DNA amounts have been estimated for *P. cumbrae* and *P. saxifrage* (2C DNA = 4.60 and 8.52 pg, respectively) by flow cytometry (FCM) (Grime and Mowforth, 1982; Suda et al., 2003; Temsch et al., 2010).

Nuclear DNA content is under strict genotypic control within the defined limits. Thus, it appears that such a variation correlates with evolutionary and systematic considerations (Bennett and Leitch, 2000; Greilhuber et al., 2005; Knight et al., 2005; Doležel et al., 2007; Bainard et al., 2013). Variation of intra/inter-specific genome size may reflect karyotypic differences, such as differences in the case of chromosome number and size (Bennett et al., 2008). Greilhuber et al. (2005) the DNA content of the unreplicated haploid chromosomal supplement, *n*, (1 *C*-value), and the amount of DNA per basic chromosome number, *x*, (1 C*x*-value, regardless of generative polyploidy, aneuploidies, or other factors). Variation in chromosome number in the *Pimpinella* genus can indicate intra- and inter-specific differences in genomic DNA quantities.

No detailed information is available regarding the DNA *C*-value, karyology, and ploidy levels of *Pimpinella* species, as cytological investigations have mainly concentrated on reporting chromosome numbers. Therefore, this research reports for the first time the karyotype criteria and genome size of 16 Iranian species of *Pimpinella*.

## Materials and Methods

### Plant Materials

**FIGURE 1:**
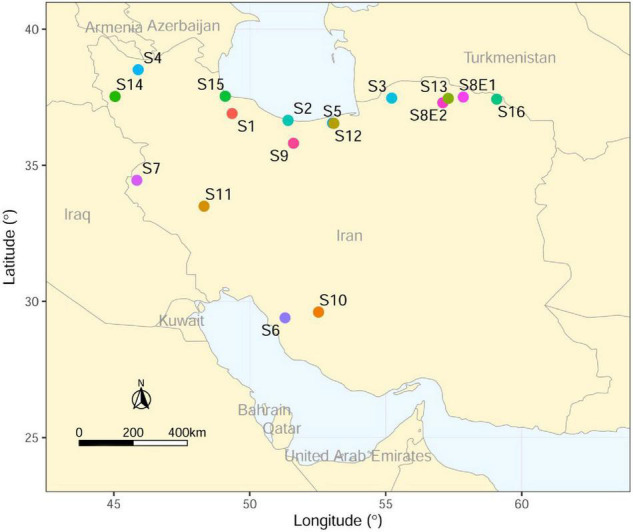
Collection sites of Iranian endemic *Pimpinella* species on the map of Iran using ArcGIS.

The seeds of 16 Iranian endemic species of *Pimpinella* were collected during the growing season in their natural habitats from different locations in Iran. Only S8 was collected from two geographical locations. The species code and geographical descriptions, including latitude, longitude, altitude (m), mean temperature (°C), and mean rainfall (mm), are shown in Table 1 and Figure 1.

**TABLE 1:** Local features of indigenous harvested Iranian *Pimpinella* species were analyzed.

Species code	Local collection sites	Latitude	Longitude	Altitude (m)	Mean Temp (°C)	Mean rainfall (mm)
*P. affinis* (S1)	Rostamabad, Gilan	36 ° 54′ N	49 ° 21′ E	1798	19.4	1337
*P. eriocarpa* (S2)	Chalous, Mazandaran	36 ° 39′ N	51 ° 25′ E	29	4	785
*P. tragium* (S3)	Gorgan, Golestan	37 ° 28′ N	55 ° 13′ E	155	17	584
*P. saxifrage* (S4)	Khodaafarin, Tabriz	39 ° 13′ N	46 ° 96′ E	400	12.5	310
*P. aurea* (S5)	Chaldoran, West Azarbaijan	36 ° 33′ N	53 ° 03′ E	2053	28	500
*P. tragioides* (S6)	Bushehr	28 ° 95′ N	50 ° 83′ E	18	25	220
*P. olivieri* (S7)	Sarpol-e-Zahab, Kermanshah	34 ° 27′ N	45 ° 51′ E	549	30	68
*P. khayyamii* (S8E1)	Esfarayen, North Khorasan	37 ° 31′ N	57 ° 51′ E	1249	14.9	186
*P. khayyamii* (S8E2)	Ghahremanabad, North Khorasan	37 ° 20′ N	57 ° 60′ E	1800	14.9	186
*P. kotschyana* (S9)	Lavasanat, Tehran	35 ° 49′ N	51 ° 37′ E	1700	13.5	350
*P. deverroides* (S10)	Shiraz, Fars	29 ° 37′ N	52 ° 32′ E	1500	18	338
*P. olivierioides* (S11)	Khorramabad, Lorestan	33 ° 29′ N	48 ° 21′ E	1200	17.4	490
*P. anthriscoides* (S12)	Djirchal, Mazandaran	36 ° 32′ N	53 ° 09′ E	2670	15	790
*P. anisactis* (S13)	Bojnord, North Khorasan	37 ° 28′ N	57 ° 20′ E	1070	13.5	272
*P. peucedanifolia* (S14)	Urmia, West Azerbaijan	37 ° 32′ N	45 ° 04′ E	1332	12.5	450
*P. khorasanica* (S15)	Dargaz, Razavi Khorasan	37 ° 26′ N	59 ° 06′ E	479	14	350
*P. rhodantha* (S16)	Rezvanshahr, Rasht	37 ° 33′ N	49 ° 08′ E	15	16	1400

### Cytogenetic Analysis

Actively growing roots of approximately 1–2 cm were cut and pretreated in a 0.002 M solution of 8-hydroxyquinoline for 4 h at 25°C and fixed in ethanol: glacial acetic acid (3:1, v/v) for 24–36 h at 4°C. Chromosome preparations from root tip cells were performed, as described by Abdolmalaki et al. (2019). For each species, ten root tips were flooded with ice-cold water twice (5 min each time), followed by 0.01 M citrate buffer twice for 5 min. Between 1 and 1.5 mm of the root tips (meristematic parts) were then digested with a 30 μl enzyme mixture containing 1% pectolyase (Sigma P3026), 0.7% cellulose (CalBiochem219466), 0.7% cellulose R10 (Duchefa C8001), and 1% cytohelicase (Sigma C8274) dissolved in 0.01 M citrate buffer with pH 4.8 for 1 h. After digestion, meristems were washed twice with citrate buffer (5 min each time) and once with ethanol for 5 min to remove the enzyme mixture. Ethanol was changed with a 70 μl fixative solution (9: glacial acetic acid/1: absolute methanol). The root tips were carefully taped using a dissecting needle until a cell suspension formed. Seven microliters of the cell suspension were then dropped onto each glass slide in a box lined with 50% humidity; it was left to dry slowly and stored in 70% ethanol. A drop containing 1 μg/ml DAPI (4′, 6-diamidino-2-phenylindole) was added to the cell area, and a coverslip was applied. High-resolution chromosome images were taken using a Nikon A1Si Laser Scanning Confocal Microscope (Nikon Instruments Inc., Japan). Chromosome measurements and karyotypic features were studied based on five well-prepared metaphase plates from different individuals.

### Karyotype Characterization

Chromosomal parameters were determined in each metaphase plate to assess the karyotypes numerically. These parameters included long arm (L), short arm (S), and total chromosome length (TL = L + S), form chromosome percentage (F% = S/ΣTL × 100), arm ratio (AR = L/S), *r*-value (S/L), chromosome relative length (RL% = TL/ΣTL × 100), the centromeric index (CI% = S/TL), and total chromosome volume (TCV = πr^2^TL), where r for TCV parameter is the average chromosome radius. Idiograms were drawn based on the arm mean values, and chromosome types were recognized by the classification system of Levan et al. (1964). Furthermore, the following karyotypic asymmetry indices were measured: total form percentage (TF% = (∑S/∑TL)×100); Huziwara, 1962), dispersion index (DI% = (X_*CI*_ CV%)/100; Lavania and Srivastava, 1999), length of total chromatin (X = 2_∑TL_), and total chromosome length coefficient variation (CV% = A_2_ 100; Paszko, 2006), difference range of relative length (DRL = RL%max-RL%min), symmetry index (S% = (TL_*min*_/TL_*max*_) × 100), Stebbins’ (1971) classification and Romero-Zarco (1986) indexes, which are defined as the intrachromosomal asymmetry index (A_1_ = 1- $\left[\sum_{i=1}^{\text{n}}\left({\text{S}}_{\text{i}}/{\text{L}}_{\text{i}}\right)/n\right]$ and interchromosomal asymmetry index ${\text{(A}}_{2}\;=\;{\text{sd/}}_{\bar{\text{X}}})$, The mean and standard deviation are represented by x and s, respectively.

### Flow Cytometric Assessment (FCM)

The 2C-DNA content of each *Pimpinella* species was estimated using flow cytometry (FCM). Flow cytometry experiments were performed using the propidium iodide (PI) staining method. A leaf of *Solanum lycopersicum* cv. Stupick with a 2C DNA value of 1.96 picograms (pg) (Doležel et al., 1992) was used as an internal reference standard. In brief, 1 cm^2^ of leaves of each *Pimpinella* species, along with 1 cm^2^ of the young leaves of the standard, were used to nuclei isolate by chopping with a sharp razor blade in 1 mL of woody plant buffer (Loureiro et al., 2007) in a Petri dish, supplemented with 50 μg ml^–1^ propidium iodide (PI), polyvinylpyrrolidone (PVP 10) and 50 μg ml^–1^ RNase. The nuclear suspension was passed through a 30 μm mesh nylon filter and then analyzed using a Cyflow Space flow cytometer (Partec GmbH, Münster, Germany) equipped with a 532 nm green high-grade solid-state laser. For each species, 5,000–10,000 nuclei per G1 peak were measured for DNA content estimates. Five different individuals per species were analyzed using linear amplification. Histograms with a coefficient of variation (CV) lower than 3% were evaluated using the FlowJo software (Version 10.6.2, Treestar, Ashland, OR, United States). Nuclear DNA content was calculated according to the following formula: Sample 2C DNA content (pg) = (Sample G_1_ peak mean/standard G_1_ peak mean) × standard 2C DNA amount (pg). As Doležel et al. (2003) stated, picogram values were converted to megabase pairs (Mbp), in which 1 pg of DNA represents 978 Mbp.

### Statistical Analysis

The data were subjected to variance analysis (ANOVA) using the GLM procedure of the SAS software (SAS, 2003) based on a completely randomized design (CRD) with five replications for both flow cytometry and karyological data. In both cases, the normal distribution of residuals and the homogeneity of variances were approved. For mean comparisons, Tukey’s test was utilized (Seijo and Fernández, 2003). Multivariate statistical analysis (Srivastava, 2002) was carried out in the Minitab software package (Minitab 17, 2010) on standardized data (mean = 0, variance = 1). A principal component analysis (PCA) was performed based on a data matrix to estimate the participation of the karyotypic parameters in the species classification (Mirzaghaderi et al., 2010). Based on karyotypic parameters, cluster analysis was carried out using the unweighted pair-group method arithmetic mean (UPGMA) and the Euclidean distance (Abedi et al., 2015). The cophenetic correlation coefficient (r) was computed to specify the goodness-of-fit of the clusters to the original data. Dot plots of mean 2C-values and means of karyotypic TCV and *X* values were generated, reflecting the presence of 4C DNA in a metaphase cell during mitotic division.

## Results

**FIGURE 2:**
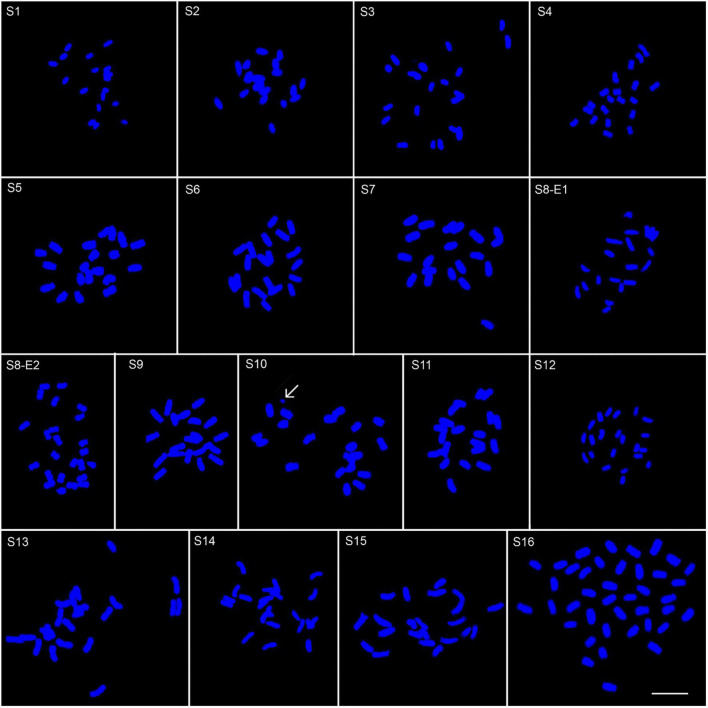
Somatic chromosomes of 17 *Pimpinella* populations of 16 species: S1 (*P. affinis*, *x* = 9), S2 (*P. eriocarpa*, *x* = 9), S3 (*P. tragium*, *x* = 10), S4 (*P. saxifrage*, *x* = 10), S5 (*P. aurea*, *x* = 10), S6 (*P. tragioides*, *x* = 10), S7 (*P. olivieri*, *x* = 10), S8 (*P. khayyamii*, E1: *x* = 10; E2: *x* = 12), S9 (*P. kotschyana*, *x* = 10), S10 (*P. deverroides*, *x* = 10), S11 (*P. olivierioides*, *x* = 10), S12 (*P. anthriscoides*, *x* = 11), S13 (*P. anisactis*, *x* = 11), S14 (*P. peucedanifolia*, *x* = 11), S15 (*P. khorasanica*, *x* = 11), and S16 (*P. rhodantha*, *x* = 20) Scale bar indicates 10 μm. The arrow shows the B-chromosome.

**FIGURE 3:**
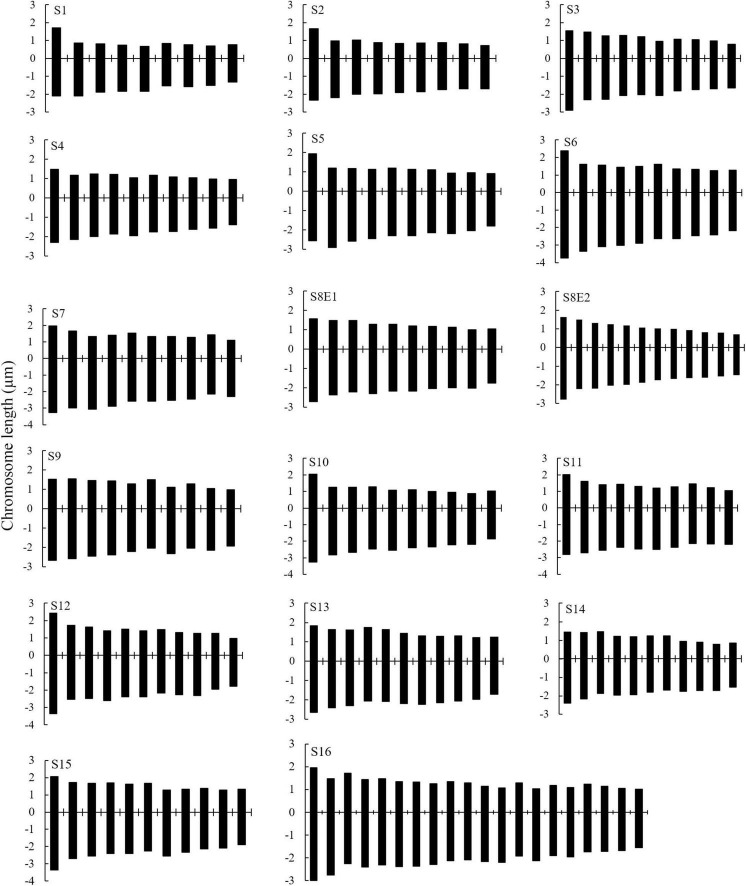
Haploid chromosome ideograms of 17 *Pimpinella* populations of 16 species. S1 (*P. affinis*, 2m + 7sm), S2 (*P. eriocarpa*, 1m + 8sm), S3 (*P. tragium*, 5m + 5sm), S4 (*P. saxifrage*, 8m + 2sm), S5 (*P. aurea*, 1m + 9sm), S6 (*P. tragioides*, 2m + 8sm), S7 (*P. olivieri*, 3m + 7sm), S8 (*P. khayyamii*, E1: 4m + 6sm; E2: 4m + 8sm), S9 (*P. kotschyana*, 5m + 5sm), S10 (*P. deverroides*, 1m + 9sm), S11 (*P. olivierioides*, 4m + 6sm), S12 (*P. anthriscoides*, 8m + 3sm), S13 (*P. anisactis*, 11m), S14 (*P. peucedanifolia*, 7m + 4sm), S15 (*P. khorasanica*, 9m + 2sm), and S16 (*P. rhodantha*, 12m + 8sm).

**FIGURE 4:**
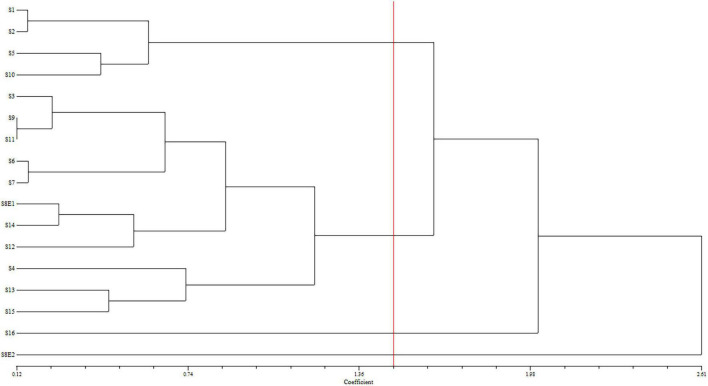
Dendrogram showing the phenetic relationships among the examined populations of *Pimpinella* of 16 species, created using the matrix of karyotype similarities with UPGMA; cophenetic correlation *r* = 0.80. S1 (*P. affinis*, *x* = 9), S2 (*P. eriocarpa*, *x* = 9), S3 (*P. tragium*, *x* = 10), S4 (*P. saxifrage*, *x* = 10), S5 (*P. aurea*, *x* = 10), S6 (*P. tragioides*, *x* = 10), S7 (*P. olivieri*, *x* = 10), S8 (*P. khayyamii*, E1: *x* = 10; E2: *x* = 12), S9 (*P. kotschyana*, *x* = 10), S10 (*P. deverroides*, *x* = 10), S11 (*P. olivierioides*, *x* = 10), S12 (*P. anthriscoides*, *x* = 11), S13 (*P. anisactis*, *x* = 11), S14 (*P. peucedanifolia*, *x* = 11), S15 (*P. khorasanica*, *x* = 11), and S16 (*P. rhodantha*, *x* = 20).

**FIGURE 5:**
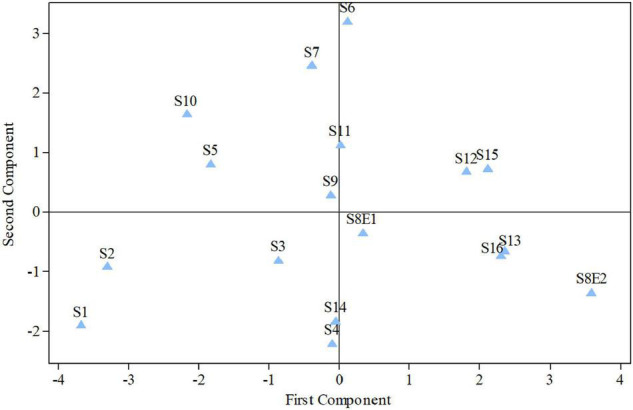
Diagram produced from the examination of the first and second components of *Pimpinella* species (strongly linked to the placement of the centromere and the length of the complements, accordingly). S1 (*P. affinis*), S2 (*P. eriocarpa*), S3 (*P. tragium*), S4 (*P. saxifrage*), S5 (*P. aurea*), S6 (*P. tragioides*), S7 (*P. olivieri*), S8 (*P. khayyamii*), S9 (*P. kotschyana*), S10 (*P. deverroides*), S11 (*P. olivierioides*), S12 (*P. anthriscoides*), S13 (*P. anisactis*), S14 (*P. peucedanifolia*), S15 (*P. khorasanica*), and S16 (*P. rhodantha*).

**FIGURE 6:**
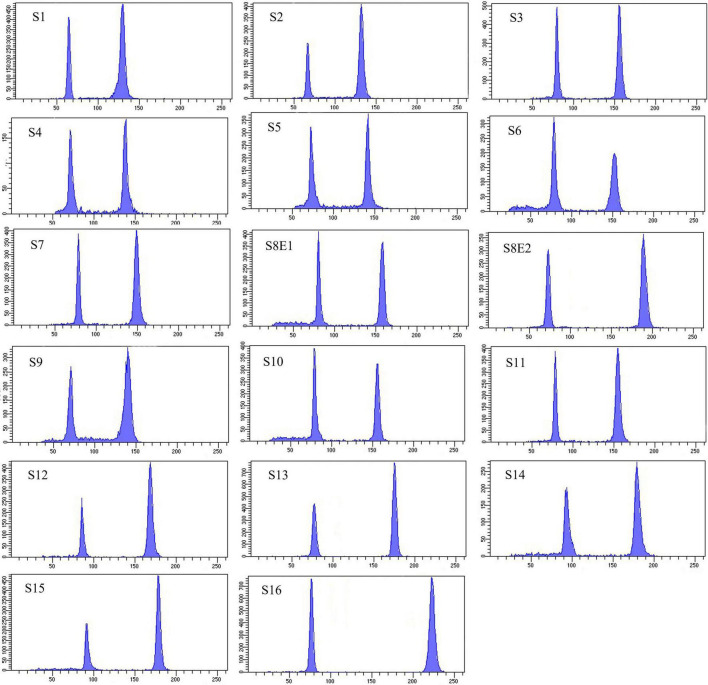
Histograms of flow cytometric 2C DNA content of 17 populations of 16 *Pimpinella* species. The left peaks refer to the *Solanum lycopersicum* cv. Stupick (2C DNA = 1.96 pg) internal reference standard and the right peaks to the *Pimpinella* samples. The *x*-axes are fluorescence intensity and the number of nuclei, respectively. S1 (*P. affinis*), S2 (*P. eriocarpa*), S3 (*P. tragium*), S4 (*P. saxifrage*), S5 (*P. aurea*), S6 (*P. tragioides*), S7 (*P. olivieri*), S8 (*P. khayyamii*), S9 (*P. kotschyana*), S10 (*P. deverroides*), S11 (*P. olivierioides*), S12 (*P. anthriscoides*), S13 (*P. anisactis*), S14 (*P. peucedanifolia*), S15 (*P. khorasanica*), and S16 (*P. rhodantha*).

**FIGURE 7:**
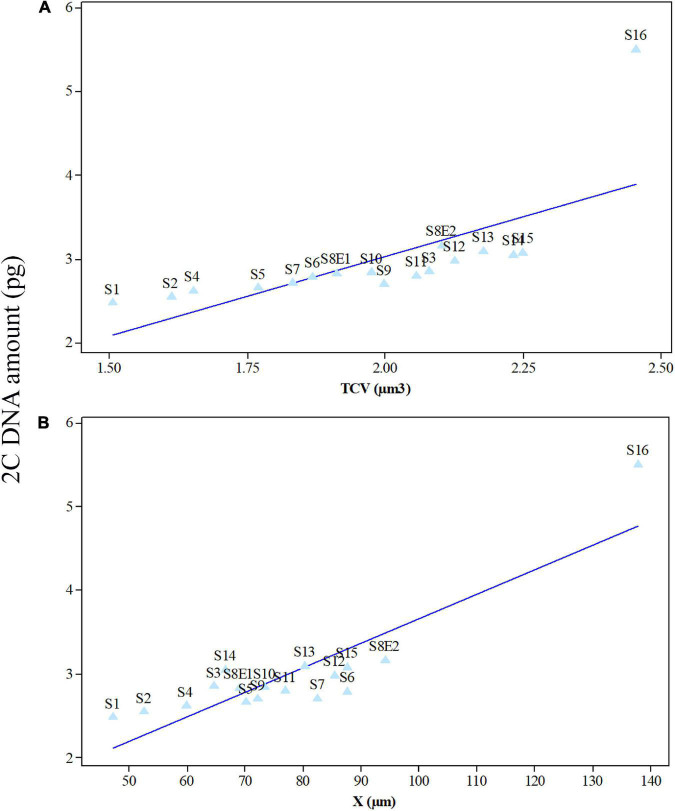
Mean leaf 2C DNA amounts (pg) of 17 *Pimpinella* populations of 16 species plotted against mean meristem total chromosome volumes (TCV, μm^3^) **(A)** and chromatin lengths (X, μm) **(B)** at mitotic metaphase. S1 (*P. affinis*), S2 (*P. eriocarpa*), S3 (*P. tragium*), S4 (*P. saxifrage*), S5 (*P. aurea*), S6 (*P. tragioides*), S7 (*P. olivieri*), S8 (*P. khayyamii*), S9 (*P. kotschyana*), S10 (*P. deverroides*), S11 (*P. olivierioides*), S12 (*P. anthriscoides*), S13 (*P. anisactis*), S14 (*P. peucedanifolia*), S15 (*P. khorasanica*), and S16 (*P. rhodantha*).

We determined the karyotypic asymmetry indices and nuclear DNA *C*-values of 17 populations from 16 different *Pimpinella* species collected from different regions of Iran. Among 17 populations examined, 16 were diploid with varying chromosome numbers, while one [*P. rhodantha* (S16)] was tetraploid. There were significant differences among the species in their long and short arms of the chromosomes, total chromosome length, *r*-value, form percentage, total chromosome volume, and centromeric index. The figure for the mitotic metaphase complements and corresponding ideograms of the studied species are shown in Figures 2, 3, respectively. In all species, the types of chromosomes were determined to be “m” (centromere at median region) and “sm,” following the chromosome nomenclature of Levan et al. (1964) (Table 2). The UPGMA grouping analysis (Figure 4) arrangement from this test is obtained with the PCA resulting species (Figure 5), which shows that the species within one cluster have the most homology in chromosomal variations. Flow cytometric data revealed a difference of 0.68 pg in 2C-value diploid populations (Figure 6 and Table 3). 2C-values were correlated with and linearly regressed upon somatic metaphase, considering either TCV or X (Figure 7).

**TABLE 2:** The karyotypic symmetry formula for *Pimpinella* species (ST: Stebbins’ type; KF: Karyotype formula).

Species	Romero-Zarco		ST	KF	DI	DRL%	CV%	S%	X	TF%
	A1	A2								
S1	0.49	0.20	3A	2m^†^ + 7sm*	6.72	7.24	19.85	55.92	47.23	33.63
S2	0.49	0.16	3A	1m + 8sm	5.30	7.61	16.30	61.35	52.48	33.56
S3	0.42	0.19	2A	5m + 5sm	6.96	6.33	19.30	55.26	64.62	36.18
S4	0.36	0.14	1A	8m + 2sm	5.48	5.09	14.30	62.56	59.77	38.55
S5	0.48	0.16	3A	1m + 9sm	5.33	5.11	15.77	60.54	70.25	33.63
S6	0.44	0.19	2A	2m + 8sm	6.72	6.32	19.06	56.71	87.70	35.10
S7	0.45	0.14	2A	3m + 7sm	4.65	5.35	13.67	64.90	82.37	34.77
S8E1	0.40	0.13	2A	6m + 4sm	4.83	3.71	12.93	65.90	68.96	36.99
S8E2	0.50	0.18	1B	6m + 6sm	6.11	7.55	17.81	58.12	94.15	35.10
S9	0.41	0.12	2A	5m + 5sm	4.46	3.55	12.13	69.85	72.20	36.72
S10	0.52	0.19	3A	1m + 9sm	6.25	8.19	19.41	54.44	73.42	32.37
S11	0.41	0.12	2A	4m + 6sm	4.58	4.11	12.45	68.02	76.88	36.67
S12	0.36	0.20	1B	8m + 3sm	7.55	7.14	19.86	47.76	85.37	38.54
S13	0.31	0.12	1A	11m	5.02	4.44	12.45	65.80	80.35	40.66
S14	0.37	0.15	2A	7m + 4sm	5.93	6.99	15.35	63.45	66.60	37.95
S15	0.34	0.15	1A	9m + 2sm	6.16	5.03	15.48	59.60	87.54	39.21
S16	0.38	0.17	2A	12m + 8sm	6.40	3.56	16.88	50.73	137.70	37.77

ST, Stebbins’ type; KF, the karyotype formula; DI, dispersion index; DRL%, difference range of relative length; CV%, coefficient of chromosome length variation; S%, symmetry index; X, total chromatin length; and TF%, karyotype total form percentage. ^†^m: centromere at the median region and *sm: centromere at the submedian region.

**TABLE 3:** 2C-value and flow cytometric DNA estimation data of *Pimpinella* species.

Species	Ploidy level	2*n*	2C-value (pg ± SE)	1C-value (pg)	1C*x*-value (pg)	Holoploid genome size (Mbp)	Monoploid genome size (Mbp)
S1	2*x*	18	2.48*^j^* ± 0.021	1.240	1.240	1212.72	1212.72
S2	2*x*	18	2.55*^hj^* ± 0.018	1.275	1.275	1246.95	1246.95
S3	2*x*	20	2.85*^de^* ± 0.018	1.425	1.425	1393.65	1393.65
S4	2*x*	20	2.62*^ghj^* ± 0.021	1.310	1.310	1281.18	1281.18
S5	2*x*	20	2.66*^fgh^* ± 0.020	1.330	1.330	1300.74	1300.74
S6	2*x*	20	2.79*^ef^* ± 0.022	1.395	1.395	1364.31	1364.31
S7	2*x*	20	2.71*^efg^* ± 0.022	1.355	1.355	1325.19	1325.19
S8E1	2*x*	20	2.83*^de^* ± 0.022	1.415	1.415	1383.87	1383.87
S8E2	2*x*	24	3.16*^b^* ± 0.017	1.580	1.580	1545.24	1545.24
S9	2*x*	20	2.70*^efgh^* ± 0.020	1.350	1.350	1320.30	1320.30
S10	2*x*	20	2.84*^de^* ± 0.021	1.420	1.420	1388.76	1388.76
S11	2*x*	20	2.80*^ef^* ± 0.032	1.400	1.400	1369.20	1369.20
S12	2*x*	22	2.98*^cd^* ± 0.022	1.490	1.490	1457.22	1457.22
S13	2*x*	22	3.09*^bc^* ± 0.025	1.545	1.545	1511.01	1511.01
S14	2*x*	22	3.05*^bc^* ± 0.021	1.525	1.525	1491.45	1491.45
S15	2*x*	22	3.08*^bc^* ± 0.021	1.540	1.540	1506.12	1506.12
S16	4*x*	40	5.50*^a^* ± 0.021	2.750	1.375	2689.50	1344.75

*According to Tukey’s test, means with different symbol letters in columns are significantly different (P < 0.01). Means with the same letter are not statistically different (P > 0.05).*

### Chromosome Numbers and Karyotype Features

Of the 16 *Pimpinella* species examined, two [*P. affinis* (S1) and *P. eriocarpa* (S2)] had chromosome numbers of 2*n* = 2*x* = 18 with a basic chromosome number *x* = 9, eight [*P. tragium* (S3), *P. saxifrage* (S4), *P. aurea* (S5), *P. tragioides* (S6), *P. olivieri* (S7), *P. kotschyana* (S9), *P. deverroides* (S10) and *P. olivierioides* (S11)] were determined as 2*n* = 20 with a basic chromosome number *x* = 10, four [*P. anthriscoides* (S12), *P. anisactis* (S13), *P. peucedanifolia* (S14), and *P. khorasanica* (S15)] were determined as 2*n* = 22, and one [*P. rhodantha* (S16)] was tetraploid (2*n* = 4*x* = 40). Interestingly, two chromosome numbers, 2*n* = 2*x* = 20 and 24, were observed in the *P. khayyamii* (S8) population.

The chromosome numbers were determined as 2*n* = 18, 20, 22, 24, and 40, with a basic chromosome number of *x* = 9, 10, 11, 12, and two diploid and tetraploid levels. The frequencies of the observed chromosome numbers were highly varied. Most of the species (52.94%) showed 2*n* = 2*x* = 20; 11.76% were 2*n* = 2*x* = 18; 23.53% were 2*n* = 2*x* = 22; 5.88% were 2*n* = 2*x* = 24; and 5.88% of the species were 2*n* = 4*x* = 40. The sporophytic chromosome numbers (2*n*) and karyotypic details for the studied species are presented in Table 2.

**FIGURE 8:**
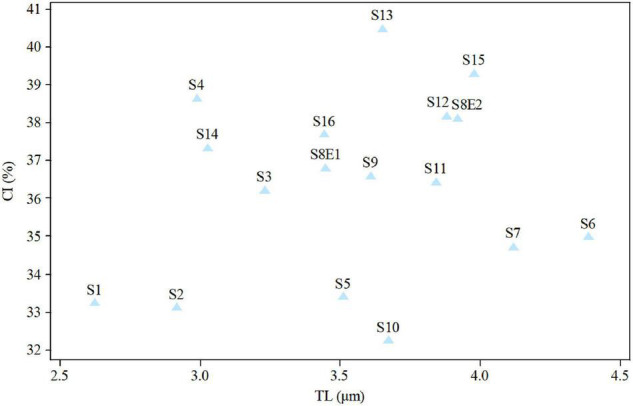
Relationship between the total length of haploid complement (TL) and centromeric index (CI) of 17*Pimpinella* populations of 17 species.

The size of the short and long arms, chromosomal length, *r*-value, TF %, total chromosome volume (TCV), arm ratio (AR), and centromeric index (CI) differed significantly across the species. The average chromosomal length varies from 2.62 μm (S1) to 4.38 μm (S6), and the haploid genome length varies from 23.62 μm (S1) to 43.85 μm (S6). The CI mean of the diploid supplement ranged between 33.10% (S2) and 40.0 (S13). In the tetraploid species (*P. rhodantha*), the mean TL, the haploid genome length, and CI were 3.44 μm, 68.85 μm, and 37.68%, respectively. Considering mean values, species with *2n* = 18 have the smallest TL and CI. The species with 2*n* = 20 have an intermediate CI but the highest TL (3.64 μm), whereas those with 2*n* = 22 have the highest CI but a TL similar to that of 2*n* = 20. Figure 8 depicts the TL and CI correlations for each species. Using Levan et al. (1964) chromosome classification, two chromosome types of “m” (The centromere is located in the central region.) and “sm” (The centromere is located in the subcentral region.) formed 14 different karyotypic formulas. Table 2 lists the various types and numbers of karyotypic formulas/species. According to different karyotypic symmetrical indexes tested, the *Pimpinella* species studied showed various symmetrical karyotypes. The greatest TF percentage value was found in S13 (40.66 %, the most symmetric), while the lowest was found in S10 (32.37%, the most asymmetric). However, the highest value of S% was identified in S9 (69.85%, the most symmetric), while S12 had the lowest value (47.76%, the most asymmetric). The highest and the lowest values of DRL% were distinguished in S10 (8.19%, the most asymmetric) and S9 (3.55%, the most symmetric), respectively. The highest and the lowest values of CV% belonged to S12 (19.86%, the most asymmetric) and S9 (12.13%, the most symmetric), respectively. Similar to the CV percent results, the greatest DI value was observed in S12 (7.55 %, the most asymmetric), while the lowest value was found in S13 (5.02%, the most symmetric) (Table 2). In conclusion, four (S%, DRL%, CV%, and DI%) among the five karyotypic symmetrical groups tested confirmed that among all 17 *Pimpinella* populations examined, S12 and S9 appear to have the most asymmetrical and symmetrical karyotypes, respectively.

**FIGURE 9:**
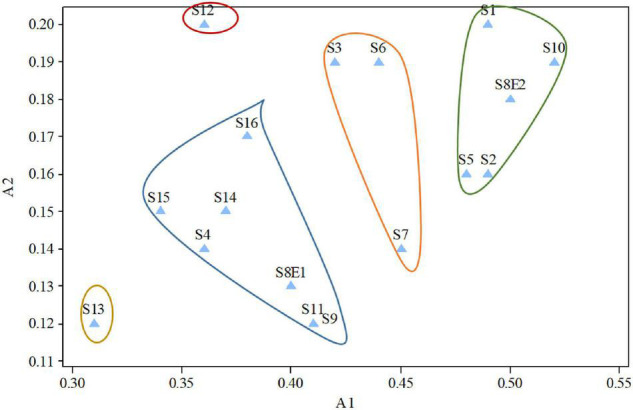
Two-dimensional graphic of the Romero-Zarco asymmetry indices (A1: intrachromosomal asymmetry index, A2: interchromosomal asymmetry index) of 16 species (17 population) *Pimpinella* species. S1 (*P. affinis*), S2 (*P. eriocarpa*), S3 (*P. tragium*), S4 (*P. saxifrage*), S5 (*P. aurea*), S6 (*P. tragioides*), S7 (*P. olivieri*), S8 (*P. khayyamii*), S9 (*P. kotschyana*), S10 (*P. deverroides*), S11 (*P. olivierioides*), S12 (*P. anthriscoides*), S13 (*P. anisactis*), S14 (*P. peucedanifolia*), S15 (*P. khorasanica*), and S16 (*P. rhodantha*).

Three species (S4, S13, and S15) were classified as 1A; eight species (S3, S6, S7, S8E1, S9, S11, S14, and S16) were classified as 2A; four species (S1, S2, S5, and S10) were classified as 3A; and the karyotype of two species (S12 and S8E2) was classified as 1B, according to the Stebbins classification (Stebbins, 1971; Table 2). The four groups of species are shown in the scatter plot of the A1 and A2 asymmetry indices: (1) taking into account one species (S13) with the most symmetrical karyotypes (A1 and A2 average = 215), (2) comprising seven species (S4, S8E1, S9, S11, S14, S15, and S16) with the symmetrical karyotypes (A1 and A2 average = 0.260), (3) including three species (S3, S6, and S7) with the asymmetrical karyotypes (A1 and A2 average = 0304), and (4) including five species (S1, S2, S5, S8E2, and S10) with the most asymmetrical karyotypes (A1 and A2 average = 0337, Figure 9). S12 falls between the second and third groups. Both indices differentiate S13 from other species, and the A1 index discriminates species by base chromosome number.

Other interesting peculiarities were found in the meta-phase plate of *P. deverroides* (S10). The chromatin body of Figure 2 could account for one acentric fragment, with the chromatids lying parallel through their length. However, another acentric fragment – corresponding to the broken chromosome end of the other fused chromosome – should also be visible, but in this case, it is not. Hence, another explanation for this chromatin body would be that it is a B-chromosome.

The UPGMA dendrogram – constructed using the matrix of karyotype similarities (Figure 4) – displayed four major clusters. The first cluster is comprised of S1, S2, S5, and S10, distinguished by the shortest complements but high RL and AR. In this cluster, species with 18 chromosomes form a subgroup. The second cluster comprises 11 species with both 20 and 22 chromosomes, which are characterized by a high L, F%, and TL. The S16 species, with 40 chromosomes with the lowest F%, form the third cluster. The fourth cluster contained S8E2, characterized by a high S, CI%, *r*-value, and the lowest AR.

The first two PCs in the karyotypic parameter’s principal component analysis (PCA) account for 80.8% of the cumulative variation, and they were shown in a 2-dimensional image (Figure 5). The first component (50.9%) emphasizes the position of the centromere, while the second component (29.9%) accentuates variation in complement length. The resulting species arrangement from this test entirely matches with those obtained from the UPGMA clustering method.

Seventeen populations of 16 *Pimpinella* species were analyzed using FlowJo software version 10.6.2 to calculate the amount of DNA in the nucleus. The acquired histograms for estimating the nuclear DNA content of each species contained two peaks; the right peaks refer to the known internal reference standard (*Solanum lycopersicum* cv. Stupick), and the left peaks refer to the unknown *Pimpinella* species (Figure 6). Table 3 shows DNA content (pg) and the genome sizes (Mbp) of studied *Pimpinella* species. The 2C-value varied from 2.48 to 5.50 pg (relating to a diploid *P. affinis* and a tetraploid *P. rhodantha*, respectively). Among the 16 diploids with chromosome numbers ranging from 18 to 24, a distinction of 0.68 pg in 2C-value (2.48 and 3.16) was observed (Table 3), while the mean 2C-value of a *P. rhodantha* (2*n* = 40, 5.50 pg) was determined to be precisely double the value of the two diploids with *x* = 10 (2*n* = 20, 2.75 pg). Tukey’s test revealed a significant difference in the 2C-value among the 16 diploid species. Meanwhile, the holoploid genome size ranged from 1212.72 Mbp (diploid S1) to 1511.01 Mbp (diploid S13), with a difference of 298.29 Mb, whereas the tetraploid haploid genome size was 2870.43 Mbp. Significant correlation was observed between chromosomal features measured, viz, X, and TCV (*r* = 0.879** and 0.701**, respectively, in Figures 7A,B), with 2C-values demonstrating linear relationships (*b* = 0.034** and 1.897**, in Figures 7A,B, respectively).

## Discussion

The findings of this study provide precise photos of the chromosomal characteristics of indigenous Iranian *Pimpinella* species for the first time. There is little information available on the chromosome numbers. The karyotype data of the studied species and information on the 2C DNA amount data were utterly insufficient. The findings of the present study revealed two ploidy levels: diploids (2*x*) and tetraploids (4*x*), as well as four different chromosomal numbers: 18, 20, 22, and 40. In prior research, such chromosome counts of 18 (Yurtseva, 1988; Pimenov et al., 2003), 20 (Al-Eisawi, 1989; Verlaque and Filosa, 1992; Pimenov et al., 1996), 22 (Constance and Chuang, 1982; Abebe, 1992; Castro and Rossello, 2007), 40 (Daushkevich et al., 1995), and ploidy levels have also been reported for other species of the *Pimpinella* genus. Furthermore, *P. affinis* was found to have different chromosome numbers of 16 and 18 (Jurtseva, 1988), *P. tragium* had 20 and 22 (Galland, 1988), *P. eriocarpa* showed 16 (Al-Eisawi, 1989), *P. rhodanth* displayed 18 and 20 (Daushkevich et al., 1995), and *P. saxifrage* showed 2*n* = 18, 20, 36, and 40 (Gawlowska, 1967).

The differences in chromosome number and chromosome morphology found among species indicate that chromosome structural changes may be used to distinguish species that are very similar to each other and cannot be separated using morphological characters. Among these species, *P. khorasanica* differs from *P. anisactis* due to the presence of two “sm” chromosome types and possesses a shorter total chromatin length. These relative differences could be used for breeding programs by facilitating chromosome identification in hybrid populations and derivatives in *Pimpinella*. Parent combinations with relative differences in the chromosome numbers/type due to chromosome pairing could result in a successful cross (Hamidi et al., 2018; Akbarzadeh et al., 2021). Three species – S3 (*P. tragium*), S13 (*P. anisactis*), and S15 (*P. khorasanica*) – are very similar to each other from the viewpoint of anatomy, and we are not able to separate them with anatomical features. In addition, they are morphologically similar to each other. Therefore, the findings of this study can be considered as a guide to separating these species, especially S13 (*P. anisactis*) and S15 (*P. khorasanica*), which are distributed in a small area in Khorassan, where they are endemic. Taxonomic criteria, including chromosome number and asymmetric indices, have been used in plant phylogenetic and taxonomic consideration of the genus (Hesamzadeh Hejazi and Ziaei Nasab, 2010).

Among species with 22 chromosomes, *P. anthriscoides* can be clearly distinguished from other species (*P. khorasanica*, *P. peucedanifolia*, and *P. anisactis*) by different evolutionary karyotype classification and karyotype formulas. This species displayed a lower 2C-value in comparison with other species with the same number of chromosomes. Also, *P. anthriscoides* differs from other species of the genus *Pimpinella* in some morphological traits, such as plant height, tepal and leaf area, and the size of reproductive organs (data not shown). Based on these observations, *P. anthriscoides* is introduced as a separate species in the distinct genus *Pseudopimpinella* in this report. Changes in chromosome morphology and genome size have been developed and used as the basic mechanisms in plant taxonomy and phylogenetic consideration of the genus (Bernardos et al., 2003; Navarro et al., 2004; Arslan et al., 2012).

To the best of our knowledge, there has been no cytological report of the presence of the B-chromosome in *Pimpinella* species. Hence, this study is the first report in this genus. The B-chromosomes are extra chromosomes and smaller than the usual A-chromosomes, of which the origin and functions are not well known (Palestis et al., 2004; Pellicer et al., 2007). The presence of B-chromosomes has been reported in plant taxa (Fregonezi et al., 2004; Felix et al., 2011; Abedi et al., 2015), and they are not necessarily for the survival of the species; however, they may act in either a positive or negative role as an adaptive function or parasitic genome, respectively (Pellicer et al., 2007; Jones, 2012). The recognition of the B-chromosome in a few individuals may favor the hypothesis of a parasitic B-chromosome (Felix et al., 2011). In the present study, the persistent presence of a B-chromosome in all examined individuals of S10 seems to support the hypothesis of an adaptive function of the B-chromosome.

Another interesting novel finding in this study was the first record of 24 chromosomes in an Iranian *P. khayyamii* (S8E2). So far, no other *Pimpinella* species have been identified with this chromosome number. Interestingly, the S8E2 *Pimpinella* population had four more chromosomes than the other diploid Iranian endemic *Pimpinella* population (S8E1; Esfarayen, North Khorasan, Iran, Figure 1), which was collected from the same geographic regions with only small differences in longitude and latitude. Previous research and our findings may lead us to conclude that the instability in both chromosome number and ploidy levels in studied *Pimpinella* species is probably due to interspecific hybridization and polyploidization, which, in turn, induces a cascade of subsequent genomic rearrangements. Above all, epigenomic rearrangements (Maletskii, 2004) may also lead to epigenetic silencing (Scheid et al., 1996). These rearrangements may increase the adaptive capacity of certain species.

According to the chromosomal parameters measured in the current study, the two diploid species with 18 chromosomes, the nine with 20 chromosomes, and the four with 22 chromosomes demonstrated intra- and inter-specific variation in X, TL, and TCV. Considering the primary chromosome parameter, S6 – which was geographically isolated from other species – appears to exhibit the largest chromatin length (X) among other *Pimpinella* species, either diploids with different chromosome numbers or tetraploids. This might indicate that chromosomal length is affected by geographical and environmental adaptability. In spite of the observed intra- and inter-specific variation, the bulk of karyotypic symmetrical indices suggests that most *Pimpinella* species, including diploids and tetraploids, possess symmetric and primitive karyotypes, which is most likely due to inter/intra hybridization and polyploidization. Thus, their similar karyotype structure causes their tendency toward crosses and does not cause a disturbance in reproduction. In general, it is believed that asymmetric karyotype can be linked to the evolutionary history of a particular group of plants (Stebbins, 1971). The high value of the A1 index (variable between 0 and 1) is considered a specialized adaptation, whereas the interchromosomal asymmetry index (A2, variable between 0 and ∞) is associated with the relative taxonomic distance between species of different taxa (Romero-Zarco, 1986). The species *P. anisactis* (S13) had the smallest A1 and A2 index values, which are probably attributable to its strong adaptability to its habitat conditions; it is therefore not particularly specialized. Three species, S1, S10, and S8E2, had the largest values of the A1 and A2 indices, indicating that these may be well-specialized species.

According to our results, FCM was effectively conducted to analyze ploidy level stability of species (Wyman et al., 1992). Different Iranian *Pimpinella* species were separated based on their nuclear DNA content, indicating inter/intraspecies diversity and confirming the cytological findings. Variability in DNA C−values is a prerequisite for use as a taxonomic character (Ellul et al., 2002). Previously, there was only a single report of the 2C-value in the diploid *P. saxifrage* (2C DNA = 8.52 pg, Temsch et al., 2010). However, this value differs considerably from our data. The reason for this is unknown but could arise from the cell cycle, rate of cell division, radiation sensitivity, ecological demeanor in plant societies and life forms, and differences between methods of DNA content analysis (Bennett et al., 2000). A surprising finding related to the 2C value is that the two diploid *P. khayyamii* accessions (S8E1, S8E2) with two different chromosome numbers of 2*n* = 2*x* = 20, 24 and two different 2C DNA content were gathered from the same area (North Khorasan) with a slight difference in geographical coordinates (Table 1). A major question remains unanswered: what causes *P. khayyamii* from the same geographic regions to differ in four chromosomes? Are there any genetic or ecological attributes that lead to this difference? Systematic investigation into different aspects of this needs to be undertaken.

The mean comparison of 2C value/chromosome between 16-diploids (0.138 pg) and only tetraploid (0.137 pg) was not substantially different (*t*-value = 0.00032; *P*-value = 0.74). For monoploid genome size, a similar conclusion was obviously true (13,811 Mbp for diploids, 1344.75 Mbp for tetraploid; *t*-value = 1.470; *P*-value = 0.163). In other words, our finding indicated that the 2C DNA-value mean and 1C*x* genome size mean in the examined *Pimpinella* species were not proportional to ploidy level and the nuclear DNA content per basic chromosome set (1 C*x*) tended to decrease when a high ploidy level was observed. A broad analysis of the mean basic genome size at different ploidies in angiosperms showed that, while basic genome size decreased with increasing ploidy, those with larger mean genome sizes at the diploid level showed a greater reduction than those with smaller mean genome sizes (Kellogg and Bennetzen, 2004; Leitch and Bennett, 2004). Studying 67 *Artemisia* species with different ploidy levels showed that 1C*x* genome size tended to decrease significantly in polyploids compared with diploids (Pellicer et al., 2007). Exceptions to this are found in the bromegrass germplasm accessions, where only a slight reduction of DNA content was detected as the ploidy level increased (Tuna et al., 2001). Different patterns of genome size variation linked to different kinds of evolutionary mechanisms and the nature of speciation/polyploidization have been shown in previous studies (Bennetzen and Kellogg, 1997; Soltis et al., 2003). Genome size reduction mechanisms, along with allopolyploidization in *Aegilops*, could be an obligatory adaptation in polyploid genome evolution (Ozkan et al., 2003). Hence, polyploidy is one possible contributor to *C*-value variation, but the relationship between *C*-value and ploidy is not straightforward (Leitch and Bennett, 2004; Murray et al., 2005). In this study, there is no relationship between the 2C DNA content of tetraploids and diploids in *Pimpinella*. Our karyotypic data on *Pimpinella* species showed a karyotype formula of 6m + 4sm for diploid S8E1 and 6m + 6sm for S8E2. In the karyotype of S8E1, metacentric “m” chromosomes were predominant, and S8E2 varied in the two types of submetacentric “sm” chromosomes. This may help us to deduce that the newly reported 24 chromosomes diploid *Pimpinella* tends to have a different evolutionary karyotype classification from the “2A” Stebbins karyotype category (relative symmetric karyotype) for most diploids to the “1B” relatively asymmetric karyotype.

In *Pimpinella* species, the significant positive correlation between the 2C-value and some karyotypic features, including the ploidy level, the total length of chromatin, and total chromosome volume, indicates that changes in nuclear DNA content have accompanied chromosome structural changes. In agreement with our finding, such a relationship between 2C-value and chromosomal parameters has been reported in *Tulipa* (Abedi et al., 2015); *Lathyrus* (Karimzadeh et al., 2011), *Thymus* (Mahdavi and Karimzadeh, 2010), and *Helichrysum* (Azizi et al., 2014).

Interestingly, in nine species with 20 chromosomes, the 1C*x* genome size of S4 (1C*x* = 1281.18 Mbp) was considerably lower (8.07%, *P* < 0.05) than the other diploid S3 (1393.65 Mbp). The average 1C*x* genome size of S3 was more than that of either diploids or tetraploids (S16, 1C*x* = 1344.75 Mbp), giving us an expanded view of variation among these species. Such considerable variability could be attractive for either polyploid induction or hybrid production among types of studied *Pimpinella* species. As a result, genome size might be utilized as an effective marker for detecting hybrids (Ellul et al., 2002).

Further work, such as fluorescence *in situ* hybridization (FISH), using repetitive sequences and rDNA genes and C-banding complementary assessment of karyology and cytology in meiosis would add more data to *Pimpinella* taxonomic studies. Our data, in combination with additional data on *Pimpinella* species worldwide, would help to recognize the origin and evolution of this genus and help to protect the endemic and threatened species with different ploidy levels and chromosome numbers. Moreover, knowledge about genome size is helpful in demonstrating any relationship between nuclear DNA content and the ecological niches of *Pimpinella* species.

## Data Availability Statement

The original contributions presented in the study are included in the article/supplementary material, further inquiries can be directed to the corresponding author/s.

## Author Contributions

SM, GM, JB, and GR conceived and designed this study. SM and AS-E conducted the experiments. SM and MH analyzed the data. SM wrote the manuscript. HN-Z and DE revised the manuscript. All authors have read and approved the published version of the manuscript.

## Conflict of Interest

The authors declare that the research was conducted in the absence of any commercial or financial relationships that could be construed as a potential conflict of interest.

## Publisher’s Note

All claims expressed in this article are solely those of the authors and do not necessarily represent those of their affiliated organizations, or those of the publisher, the editors and the reviewers. Any product that may be evaluated in this article, or claim that may be made by its manufacturer, is not guaranteed or endorsed by the publisher.
